# Correlates of mental health in the Swiss Household Panel – a network analysis

**DOI:** 10.1093/eurpub/ckac131.475

**Published:** 2022-10-25

**Authors:** D Gondek, E McElroy, L Bernardi

**Affiliations:** 1 University of Lausanne, Lausanne, Switzerland; 2 Ulster University, Ulster, UK

## Abstract

**Background:**

Firstly, we aim to describe any differences in the mean levels of correlates and indicators of mental health and wellbeing between young (25-39 years) and middle-aged adults (40-55 years). Secondly, we aim to compare the network models depicting interrelations between correlates and indicators of mental health and wellbeing among these age groups.

**Methods:**

This paper draws on longitudinal data from 6 waves (2013-2018) of the Swiss Household Panel (SHP) study, with a total sample of 5,315 individuals, including 2,044 young (25-39 years) and 3,271 middle-aged (40-55 years) participants. We used network analysis to examine and present complex relationships between the correlates and the indicators of mental health and wellbeing.

**Results:**

Middle-aged individuals had worse mental health and wellbeing on all indicators but energy and optimism, which did not differ across groups. The effect sizes (according to Cohen’s d) were small, reaching the maximum of 0.20 for sadness. Despite higher household income and financial satisfaction, perceived job insecurity and work strain were higher in midlife, with socioeconomic prestige being lower. Moreover, middle-aged individuals had lower social support, relationships satisfaction, and health satisfaction. The network was denser in midlife, with two direct interrelations being stronger in this age group: health satisfaction and energy/optimism as well as accommodation satisfaction and life satisfaction. There were also several other differences in indirect interrelations between correlates and indicators of mental health and wellbeing, including a potentially more important role of self-mastery in midlife in bridging socioeconomic indicators, wellbeing and mental health.

**Conclusions:**

We suggest further exploring the workplace as an avenue to improving population mental health and wellbeing, with a particular focus on the role of self-mastery.

**Key messages:**

• Middle-aged individuals appear to have worse mental health and lower wellbeing than young adults.

• Health satisfaction is not only lower in midlife, but it seems of greater importance for mental health and wellbeing.

